# Repurposing Potential of the Antiparasitic Agent Ivermectin for the Treatment and/or Prophylaxis of COVID-19

**DOI:** 10.3390/ph15091068

**Published:** 2022-08-27

**Authors:** Hoda Awad, Basmala Hassan, Sara Dweek, Yasmeen Aboelata, Mutasem Rawas-Qalaji, Iman Saad Ahmed

**Affiliations:** 1 Department of Pharmaceutics & Pharmaceutical Technology, College of Pharmacy, University of Sharjah, Sharjah 27272, United Arab Emirates; u19104592@sharjah.ac.ae (H.A.); u17100019@sharjah.ac.ae (B.H.); u17103078@sharjah.ac.ae (S.D.); u17102409@sharjah.ac.ae (Y.A.); mqalaji@sharjah.ac.ae (M.R.-Q.); 2 Research Institute of Medical and Health Sciences, University of Sharjah, Sharjah 27272, United Arab Emirates

**Keywords:** ivermectin, COVID-19, SARS-CoV-2, coronavirus, drug repurposing

## Abstract

Due to the rapid, vast, and emerging global spread of the Coronavirus Disease 2019 (COVID-19) pandemic, many drugs were quickly repurposed in a desperate attempt to unveil a miracle drug. Ivermectin (IVM), an antiparasitic macrocyclic lactone, was tested and confirmed for its in vitro antiviral activity against severe acute respiratory syndrome coronavirus-2 (SARS-CoV-2) in early 2020. Along with its potential antiviral activity, the affordability and availability of IVM resulted in a wide public interest. Across the world, trials have put IVM to test for both the treatment and prophylaxis of COVID-19, as well as its potential role in combination therapy. Additionally, the targeted delivery of IVM was studied in animals and COVID-19 patients. Through this conducted literature review, the potential value and effectiveness of the repurposed antiparasitic agent in the ongoing global emergency were summarized. The reviewed trials suggested a value of IVM as a treatment in mild COVID-19 cases, though the benefit was not extensive. On the other hand, IVM efficacy as a prophylactic agent was more evident and widely reported. In the most recent trials, novel nasal formulations of IVM were explored with the hope of an improved optimized effect.

## 1. Introduction

In March 2020, the World Health Organization (WHO) declared COVID-19 a pandemic, and a global emergency following its widespread from Wuhan City, China, to the rest of the world [1]. This was the first designation since the H1N1 influenza pandemic in 2009. The identified cause behind the disease is a novel coronavirus called severe acute respiratory syndrome coronavirus-2 (SARS-CoV-2). Two years later and as of June 2022, COVID-19 has affected more than 500 million people worldwide, costing more than 6 million lives [2]. Among the highly pathogenic coronaviruses, SARS-CoV-2 was found to be one with the most extensive spread in epithelial cells of the upper respiratory tract [3].

The pathogenesis of SARS-CoV-2 is initiated by viral entry through the angiotensin-converting enzyme 2 (ACE-2) receptor in the host cell. After entry, RNA is translated into viral proteins upon its release in the cytoplasm. The resulting symptoms and clinical manifestations include fever, headache, myalgia, and respiratory symptoms [4]. Some host-related factors can increase the risk and severity of symptoms, which may include older age and male gender, as well as co-existing medical conditions and impaired immunity [5]. Throughout the past two years, several prominent variants have emerged including Alpha, Beta, Delta, and the latest and most infectious, Omicron [4].

Consequently, several drugs have been and continue to be repurposed for the prophylaxis and treatment of COVID-19 [6,7]. These drugs include antimalarials, such as chloroquine phosphate, hydroxychloroquine (HCQ), and antivirals such as lopinavir/ritonavir, umifenovir, remdesivir, and favipiravir. Moreover, monoclonal antibodies, such as tocilizumab, are being investigated as potential COVID-19 treatments [8]. Interestingly, the antiparasitic macrocyclic lactone ivermectin (IVM), due to its in vitro antiviral activity, became a research target for its potential against COVID-19 [9].

## 2. Background

**Figure 1:**
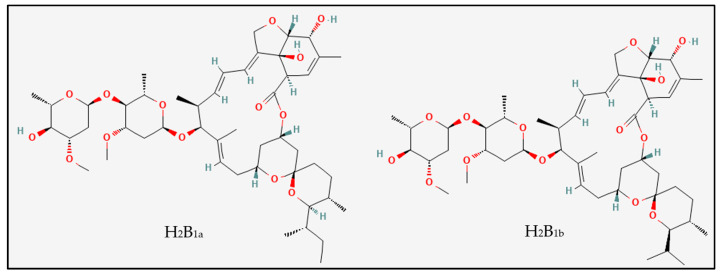
Chemical structure of Ivermectin (IVM), consisting of components H_2_B_1a_ (C_48_H_74_O_14_, 875.1 g/mol) and H_2_B_1b_ (C_47_H_72_O_14_, 861.1 g/mol). Figure adapted from references [18,19].

Ever since its discovery in 1975 [10,11], IVM has always attracted attention, starting with a Nobel Prize and ending with what could potentially be a solution to the current world pandemic. It belongs to the naturally occurring family of avermectins produced by the bacterium *Streptomyces avermitilis* [10]. Its broad antiparasitic spectrum is achieved through the hyperpolarization of the invertebrate’s cell membrane, with subsequent parasite paralysis [9]. In 1981, it came into use for the treatment of multiple indications, including strongyloidiasis, onchocerciasis (river blindness) [9,12], ascariasis [13], scabies [14], cutaneous larva migrans [15], filariasis [9,16], lice and myiasis [17]. Through its wide applications, affordability, high efficacy, and safety, the wide therapeutic usefulness of IVM resulted in its inclusion in the 21st Essential Medications List by the WHO. The chemical structure of IVM is shown in Figure 1 [18,19].

## 3. Pharmacokinetics of IVM

Oral, topical, and injectable dosage forms of IVM are available; however, only the oral and topical formulations are licensed for administration in humans. Oral IVM is rapidly absorbed, with similar rates of absorption for both solid and liquid dosage forms but varying systemic bioavailability (twice for ethanolic solution compared to capsules/tablets) [20]. Upon administration of a standard oral dose (a single dose of 150–200 μg/kg) [21], maximum plasma concentrations are reached within three to five hours. A second peak is often observed indicating enterohepatic recycling occurring [22].

Due to its high lipophilicity, IVM has a large volume of distribution (V_d_). Its central compartment V_d_ is 3.1 to 3.5 L/kg in healthy adults [20]. The drug is highly distributed in fats but has low distribution in the subcutaneous fascia [22]. Plasma protein binding was found to be 93% with preferential binding to serum albumin, which results in higher levels of free drug in populations with hypoalbuminemia and malnutrition [23]. Despite its wide distribution, IVM does not distribute to the cerebrospinal fluid [20].

The metabolism of IVM is hepatic, with cytochrome P-4503A4 being the main responsible isoform. The drug is metabolized into 10 compounds mostly through hydroxylation and demethylation [24]. The elimination half-life of IVM was reported to be 18–24 h; however, its pharmacological antiparasitic activity was reported to be sustained for up to months. Its excretion is primarily fecal with less than 1% excreted in the urine [20].

In this review article, the potential use of IVM in the therapy and prophylaxis of COVID-19 will be discussed. Amid the pandemic, many clinical trials and studies were conducted across the world to test the efficacy of IVM in the treatment and prophylaxis of COVID-19. Its affordability and global availability could have potentially played a major role for its selection in these clinical trials and studies, specifically the ones performed in developing countries. This was evident, as most of the trials were held in countries such as Egypt, India, Argentina, Mexico, Brazil, and Bangladesh. Despite the rise in its investigative trials, IVM is not yet approved by most of the regulatory authorities for its use in COVID-19 patients nor is it added to hospitals’ treatment protocols.

## 4. IVM Potential Mechanism of Action against COVID-19

**Figure 2:**
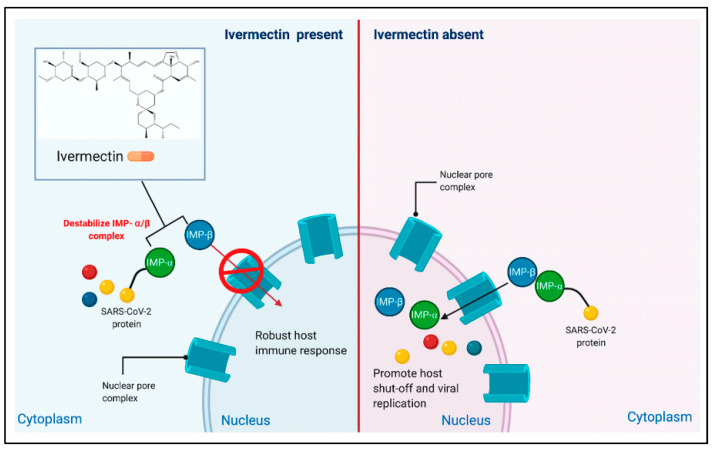
Potential antiviral action of IVM inhibiting IMPα/β1-mediated nuclear import of viral proteins of SARS-CoV-2. Figure adapted from reference [26] (CC BY 4.0).

Different hypothesized mechanisms of action were explored in multiple studies to understand the IVM activity against COVID-19. In March 2020, Caly et al. introduced IVM as a potential therapy for the pandemic by demonstrating its antiviral activity against SARS-CoV-2 in Vero E6 cells. The article suggested that the antiviral activity is gained through the inhibition of importin (IMP)-α/β1-mediated nuclear import of viral proteins of SARS-CoV-2, resulting in the inhibition of RNA replication [25]. This was further illustrated by Mudatsir et al. as shown in Figure 2 [26]. It is important to note that the levels of IVM used that demonstrated inhibitory activity (5 μM) are not achievable in humans, as they are 100 times more than the standard dose (200 μg/kg). Furthermore, most of the conducted clinical trials used the standard dose with a higher frequency of administration rather than a higher dose. Caly’s study was the gateway to the hundreds of IVM-based trials which took place in 2020 and are still ongoing to this day. Yang et al. reported that in addition to the inhibition of IMP-α/β1, IVM can dissociate the previously formed IMP-α/β1 heterodimer in Vero cells [27]. Another molecular docking study revealed that IVM binds as well to dimeric 3C-like protease and non-structural protein (NSP19) in addition to IMP-α [28].

On the contrary, IVM at a concentration of 10 μM was found to fail in inhibiting SARS-CoV-2 infection in a study conducted on human airway-derived cell models. These contradicting results suggest that the previously determined IVM activity conducted in Vero cells might not correlate with the results obtained in different models, and thus, should not be used to interpret IVM activity in humans [29].

**Figure 3:**
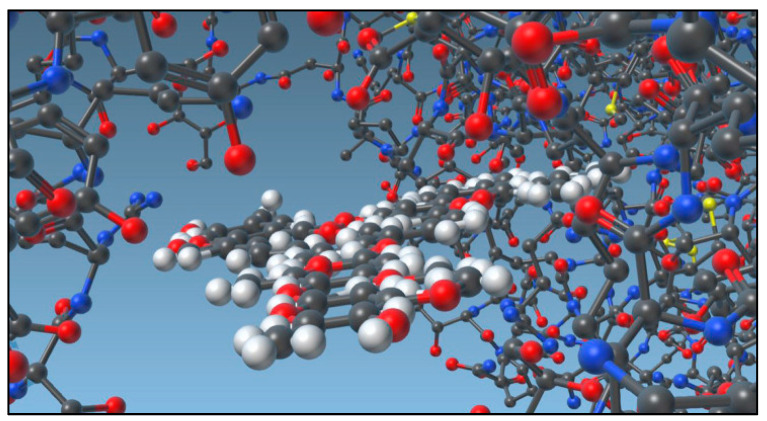
IVM bridging between SARS-CoV-2 (left) and ACE2 receptor (right). Figure adapted from reference [30] with permission from the International Institute of Anticancer Research.

Another mechanism of action for COVID-19 is through its interaction with the ACE-2 receptor. A study by Lehrer and Rheinstein showed that IVM docked leucine 91 on the spike and docked histidine 378 on the ACE-2 receptor (Figure 3) [30]. The IVM docking may obstruct the spike’s adhesion to the human cell membrane, due to the drug’s ability to act as a bridge between the virus and the receptor. Similar results were reported by Saha and Raihan, which showed that IVM has large binding affinity through hydrogen bonding to leucin 492, glutamine 493, glycine 496 and tyrosine 505 residues in the spike protein which favors binding to ACE-2 [31]. Eweas et al. found that in addition to the spike protein and ACE-2 binding, IVM can bind to type II transmembrane serine protease (TMPRSS2), which plays a role in the binding and fusion of the virus into the cell membrane. Further, binding to main protease (Mpro), papain-like protease (PLpro), nucleocapsid phosphoprotein and NSP14 of the SARS-CoV-2 prevents the post-translation processing, replication, and assembly [32].

The immunomodulatory impact of IVM through the cholinergic anti-inflammatory pathway activation was also explored in golden hamsters by de Melo et al. as a potential mechanism of action in COVID-19. The study concluded that the administered IVM single dose of 400 μg/kg injected subcutaneously resulted in the sex-dependent prevention of clinical deterioration and reduction in olfactory deficit, which was linked with a remarkable reduction in the interleukins (IL)-6/IL-10 ratio in the lung tissues [33]. These findings correlate with a significantly lower IL-6/IL-10 plasmatic ratio reported in another study for patients with mild COVID-19 infection, compared to patients requiring intensive care unit (ICU) admission [34].

## 5. IVM Role in the Treatment of COVID-19

### 5.1. Therapeutic Benefit

Many trials were conducted in different countries with the aim of exploiting the therapeutic benefit of IVM in COVID-19 cases. In a randomized, controlled, double-blinded study between May and November 2020, Babalola et al. selected 62 mild-to-moderate COVID-19 patients at the Lagos University Teaching Hospital, Lagos, Nigeria, and divided them into 3 treatment groups. Over the duration of two weeks, Group A received 6 mg intravenous IVM twice a week, Group B received 12 mg intravenous IVM twice a week, and Group C received oral lopinavir/ritonavir daily and a placebo (control group). All patients showed mild symptoms, such as cough, headache, and fever. No patients were on mechanical ventilation. Group A showed a negative polymerase chain reaction (PCR) result 3.15 days prior to the control group, while group B showed a negative PCR result 4.5 days prior to the control group (*p* = 0.0066). In this study, it was concluded that 12 mg of intravenous IVM is significantly effective, as it showed a shortened duration of treatment. No adverse side effects were documented during this trial despite using a high dose of IVM [35].

In another study, similar outcomes on PCR negativity were reported. Mohan et al. conducted a double blinded, randomized controlled study on 125 hospitalized patients with mild-to-moderate COVID-19 in the All India Institute of Medical Sciences (New Delhi, India) between July and September 2020. Forty patients received 24 mg IVM, forty patients received 12 mg IVM, and forty-five patients received a placebo. IVM oral elixir formulation was used. No serious adverse events were recorded. Although the PCR negativity at the 5th day was high in both IVM groups (47.5% for 24 mg group, 35.0% for 12 mg group) compared to the placebo group (31.1%), these results were statistically insignificant (*p* = 0.30) [36].

### 5.2. Viral Clearance/Load

The IVM effect on the viral clearance/load of the COVID-19 patients was also studied. A pilot, randomized, controlled, outcome-assessor blinded trial by Krolewiecki et al. was conducted in 4 hospitals in Buenos Aires, Argentina, between May and September 2020. The study included 30 patients receiving 600 μg/kg/day IVM via oral tablet for 5 days and 15 patients serving as the control group. Both groups also received the standard of care which at that time included the hospitalization of all symptomatic patients. The viral load was not found to be different between the two groups; however, patients with higher IVM plasma levels exhibited significant viral load reduction (72%) compared to the control group (42%, *p* = 0.004). The high dose of IVM was well tolerated [37].

Another randomized controlled trial by Samaha et al. conducted between September and November 2020 involved 100 asymptomatic COVID-19 patients in Lebanon. Fifty participants received a single oral dose of IVM based on their body weight (participants with body weight of 45–64 kg, 65–84 kg, and ≥85 kg received 9 mg, 12 mg or 150 μg/kg, respectively), and fifty participants were in the control group. Both groups also received zinc and vitamin C supplements. Viral load was measured utilizing the cycle threshold indicator (Ct-values) in which a value of 30 or higher indicates an insignificant viral load. At 72 h after starting the regimen, it was found that Ct-values in the IVM group reached 30.14 ± 6.22, compared to the control group, reaching 18.96 ± 3.26 (*p* < 0.001). Additionally, the development of clinical symptoms was more evident in the control group [38].

Another study reported an insignificant effect on the viral load, but a significant impact on the recovery speed. In Barcelona, Spain, a double-blinded randomized control trial by Chaccour et al. was designed to test the efficacy of a single maximum dose of IVM in reducing COVID-19 transmission. Patients of Clinica Universidad de Navarra with non-severe COVID-19 participated in the trial between July and September 2020. The participants were divided randomly into two equal groups (1:1). One group (n = 12) received a single oral dose of IVM (400 μg/kg), and the second group (n = 12) received a placebo within 72 h of fever or cough onset. The viral load, infectivity, as well as the number of patients with positive PCR on day 7, were the main outcomes compared between the two groups. On day 7, there was no difference in the proportion of positive PCR patients between the two groups (12/12 and 12/12). The viral loads of the IVM group for gene E and gene N on day 4 and day 7 were lower, though the difference was non-significant compared to the placebo group (*p* > 0.1 for all). IgG titers in the IVM group were also non-significantly lower (*p* = 0.24) compared to the placebo group. However, the IVM group had a faster recovery from hyposmia/anosmia (76 patient-days) compared to the placebo group (158 patient-days) (*p* < 0.001). Patient-days is the unit used to measure the number of patients occupying beds in a healthcare facility for the period for which an assessment is being conducted [39].

Earlier viral clearance was also reported by some studies. In November 2020, Ahmed et al. published a randomized, double-blinded, placebo-controlled trial in Dhaka, Bangladesh, to study the efficacy and safety of oral IVM in the management of COVID-19. Seventy-two hospitalized patients were divided equally into three treatment groups. Group 1 received 12 mg of oral IVM once daily for the duration of 5 days. Group 2 received 12 mg of oral IVM as a single dose with 200 mg doxycycline on day 1 followed by doxycycline 100 mg every 12 h for 4 days. Group 3 was the placebo group. The results of the trial showed no statistically significant difference between the three groups in the recession of clinical symptoms, including fever, sore throat, and cough. However, this was not the case with the viral clearance. Groups 1 and 2 experienced earlier viral clearance (9.7 days and 11.5 days respectively), compared to Group 3 (12.7 days). The number of days for viral clearance was significantly lower in Group 1 compared to Group 3 (*p* = 0.02), unlike Group 2 (*p* = 0.27). Overall, the five-day course of IVM showed faster viral clearance, which suggests the potential role of IVM in the management of COVID-19 [40].

### 5.3. Symptoms Resolution/Hospitalization Length

On the other hand, some studies concluded that IVM has an insignificant role in COVID-19 treatment when it comes to clinical manifestations and health state. In Mexico, Gonzalez et al. conducted a randomized, controlled, double-blinded trial measuring the length of hospitalization in severe COVID-19 patients between April and August 2020. This trial involved 106 participants who were divided into 3 groups: IVM, HCQ, and a placebo group. Group 1 was treated with oral IVM (12 mg in patients weighing <80 kg and 18 mg in those >80 kg), group 2 received HCQ 400 mg every 12 h on the 1st day, followed by 200 mg every 12 h for four days, and group 3 received a placebo. This study concluded that there was no significant difference between the three groups in the duration of hospitalization, with an average of 7 days for HCQ group, 6 days for IVM group, and 5 days for the placebo. There was also no significant difference between the three groups in the respiratory deterioration or death [41].

Another trial that took place between March and October 2020 by Abd-Elsalam et al. tested the antiviral potential of IVM compared to standard care in mild to moderate COVID-19 patients hosted in Tanta and Assiut University Hospitals, Egypt. The trial used a 1:1 randomized, open-label parallel-group design. The IVM group (82 participants) received a single oral dose of IVM tablets (12 mg/day) for 3 days, after which the Egyptian standard protocol care was added, while the control group (82 participants) received the Egyptian standard protocol care alone for 14 days. The Egyptian standard protocol included an empiric antibiotic, oseltamivir (if needed), paracetamol, oxygen, and mechanical ventilation in case of PaO_2_ (partial pressure of oxygen in arterial blood) less than 60 mmHg. The results showed a shorter hospital stay for the IVM group (8.82 ± 4.94 days) in comparison with the control group (10.97 ± 5.28 days). However, these results lacked statistical significance (*p* = 0.085). In both groups, three patients needed mechanical ventilation. The mortality rates in both groups were not significantly different with 3.7% in the IVM group, compared to 4.9% in the control group (*p* = 1.00). Overall, despite the outcomes bearing no significant difference, the study did observe a pattern of shortened hospitalization periods in the IVM group [42].

Similarly, López-Medina conducted a double-blinded randomized trial in Cali, Colombia, on 400 patients with mild COVID-19 between July and December 2020. One group of 200 patients received 300 μg/kg of oral IVM (as a solution) per day for 5 days, and 200 patients received a placebo. The difference in time to symptoms resolution was statistically insignificant between both groups (10 days in the IVM group compared to 12 days in the placebo group) [43].

Lastly, in Corrientes, Argentina, during the period between August 2020 and February 2021, Vallejos et al. carried out a randomized, double-blinded, placebo-controlled trial to determine whether the use of IVM can help with hospitalization prevention in patients with early COVID-19. The study was conducted on 501 patients. Two hundred and fifty of these patients were randomized into a weight-based dose of oral IVM for 2 days (participants with body weight of ≤80 kg, >80 to ≤110 kg, and >110 kg received 12 mg, 18 mg, or 24 mg, respectively) and the rest took placebo treatment. The results showed no significant difference in hospitalization prevention with 14 patients (5.6%) of the IVM group requiring hospitalization compared to 21 patients (8.4%) in the control group. However, hospitalized patients that used IVM required invasive mechanical ventilation earlier compared to those on placebo. Therefore, the authors concluded that there was no significant effect of IVM on hospitalization prevention in COVID-19 patients [44]. A summary of the reviewed studies on the role of IVM in the COVID-19 treatment is included in Table 1.

## 6. IVM Role in the Prophylaxis against COVID-19

Treating COVID-19 with IVM was not the only question of interest in the conducted research. Many studies were conducted and continue to investigate the potential role of IVM as a chemoprophylactic agent in COVID-19. A clinical randomized open label-controlled trial was conducted by Shoumann et al. at the University of Zagazig, Egypt, to investigate the use of IVM as prophylactic therapy in COVID-19 during June and July 2020. Three hundred four asymptomatic participants were enrolled in this study. All the participants were in close contact with confirmed COVID-19 family members. The participants were divided into two groups. The IVM group included 203 asymptomatic participants who received the first dose of oral IVM ranging between 200 and 300 μg/kg on the enrollment day and an equal second dose on the third day. In the non-intervention group, no treatment was provided to 101 asymptomatic participants. The groups were followed-up for a 2-week period for the common symptoms, complete blood count (CBC), and C-reactive protein (CRP). PCR tests were conducted after the follow-up period and showed that 59 participants (58.4%) tested positive for COVID-19 in the non-intervention group. On the other hand, only 15 participants (7.4%) tested positive in the IVM group. This study reported that there was a 2-day delay in symptoms development in the IVM group, compared to the control group. IVM protection was more noticeable in participants less than 60 years old. Differences between the two groups in terms of outcomes were highly significant (*p* = 0.001) [45].

A protection program in Itajaí, Brazil, reported by Kerr et al., investigated the role of IVM in COVID-19 prophylaxis during January 2022. This was an observational prospective study that included 159,561 citizens. They were divided into two major groups: the IVM group and the control group. The IVM group included 113,845 participants who received a dose of 200 μg/kg of IVM as a prophylactic treatment for 2 consecutive days. The control group consisted of 45,716 participants who did not receive any prophylactic agents. After 15 days of taking IVM, only 4311 (3.9%) tested positive for COVID-19, while 3034 (6.6%) were reported positive from the control group. According to the findings of this study, there was an observed reduction in the infection rate by 44%, the mortality rate by 68%, and the hospitalization rate by 67% in the IVM prophylaxis group, compared to the control group (*p* < 0.0001 for all). After this study and considering the benefit and risk analysis, the authors are waiting for the approval of using IVM by agencies throughout the world, such as the Food and Drug Administration (FDA), European Medicines Agency (EMA), and the Brazilian Health Regulatory Agency (ANVISA) [46].

Apart from the aforementioned trials, studies investigating the prophylactic role of IVM were mostly conducted in a retrospective manner. A trial was conducted in Africa investigating COVID-19 infection rates among countries which have either participated in the earlier African Program for Onchocerciasis Control (APOC) and those which did not (non-APOC). APOC was a mass onchocerciasis prevention program which began in 1989 and continued until 2015. The program distributed IVM to a total of 19 countries and treated 90 million individuals annually. The study demonstrated significantly lower COVID-19 infection rates, as well as significantly lower mortality in APOC countries, compared to non-APOC [47].

Similarly, in the period between June and July 2020, Morgenstern et al. conducted an observational retrospective cohort study in Punta Cana, Dominican Republic, to evaluate the pre-exposure prophylactic effect of IVM in 271 healthcare personnel who adhered to a weekly oral dose of 200 μg/kg of IVM versus a control group of another 271 healthcare personnel who did not adhere to such regimen. After 28 days of follow-up, the IVM exposed group showed statistically significant prophylaxis against the infection with only 1.8% of the personnel developing COVID-19 compared to 6.6% in the control group (*p* = 0.006). The results suggest that the preventive use of a weekly oral IVM dose could be an option for healthcare workers and as an adjunct to immunization [48].

Additionally, a hospital-based matched case-control study in AIIMS Bhubaneswar, India, conducted by Behera et al. from September to October 2020 on 372 individuals, showed that two oral doses of 300 μg/kg IVM, 72 h apart, taken as a prophylactic agent reduced the COVID-19 infection by 73% in healthcare workers within the subsequent month [49].

Another study by Hellwig and Maia in October 2020 explored the impact on COVID-19 patients associated with the prophylactic administration of IVM. They gathered data from countries that utilize IVM as part of their prophylactic chemotherapy (PCT) campaign and countries which do not include IVM in the PCT and compared both to countries that do not deploy PCT at all. The incidence of COVID-19 was significantly lower (*p* < 0.001) in populations that previously received IVM compared to populations without PCT, while it showed lower but statistically insignificant incidence in populations receiving PCT without IVM [50]. A summary of the reviewed studies on the role of IVM in the prophylaxis against COVID-19 is included in Table 2.

## 7. Combined Therapy of IVM against COVID-19

### 7.1. Therapeutic Benefit

The use of IVM as part of a combination therapy was reported to have a promising role against COVID-19 in multiple studies. Chowdhury et al. performed a randomized controlled trial between May and June 2020 in Bazar, Bangladesh, comparing the effects of two possible combination therapies in COVID-19 patients. The first combination was IVM with doxycycline, while the second was HCQ with azithromycin. Group A included 60 patients who received IVM 200 μg/kg as a single oral dose on day 1 of the trial with doxycycline 100 mg twice daily for 10 days, while group B included 56 participants who received HCQ 400 mg on the first day then 200 mg twice daily with azithromycin 500 mg/day for 5 days. All patients in group A showed negative PCR results within an average of 8.93 days, and symptoms relief was achieved within an average of 5.93 days, and 55.1% of the participants were symptom free by the fifth day of the trial. Out of the patients in group B, 96.36% showed negative PCR results after an average of 9.33 days, and all patients were symptom free after an average of 6.99 days. In group A, 31.67% of the patients experienced side effects of the combined therapy, compared to 46.43% in group B. The study concluded that the combination of IVM with doxycycline was superior to the combination of HCQ with azithromycin in patients with mild–moderate COVID-19 [51].

### 7.2. Viral Clearance

IVM was also reported to enhance viral clearance when utilized in combination therapy. A study conducted by Elalfy et al. at Mansoura University Hospital, Egypt, from May to October 2020 included 113 participants who tested positive for COVID-19. In this non-randomized clinical trial, the patients were divided into two groups: the white arm group which included 51 patients, and the yellow arm group which included 62 patients. The white arm group was treated with paracetamol 3 tablets per day, zinc supplements twice daily, healthy nutrition and hydration, and azithromycin, case by case. The yellow arm group was treated with multidrug therapy, including nitazoxanide, ribavirin, and IVM plus zinc. The patients received 500 mg nitazoxanide every 6 h, ribavirin 400 mg every 6 h, and a weight-based oral IVM dose every 72 h. In patients weighing between 60 and 90 kg, the IVM dose ranged between 200 and 400 μg/kg, patients weighing between 90 and 120 kg received 300–400 μg/kg IVM, while patients weighing above 120 kg received 30 mg IVM. The white arm group showed a viral clearance rate of 0% in the first week, and 13.7% on day 15. On the other hand, the yellow arm group showed a viral clearance rate of 58.1% in the first week, and 73.1% on day 15. The authors concluded that the use of the triple therapy (nitazoxanide, ribavirin, and IVM in addition to zinc) cleared the COVID-19 virus within a shorter period compared to supportive or symptomatic treatment alone. There were no reported drug-related side effects, except for minor ones, such as stomach upset and diarrhea [52].

### 7.3. Symptoms Resolution

The impact of IVM combination therapy on the clinical symptoms’ resolution was also explored. In a randomized, double-blinded clinical trial published by Shahbaznejad et al. in Mazandaran, Iran, 69 patients were divided and randomized into either an IVM-receiving group or a standard treatment group containing 35 and 34 patients respectively. The study was conducted between May and July 2020. Both groups received supportive treatment for COVID-19, which was decided according to Iranian protocols at the time (HCQ and/or lopinavir/ritonavir). The intervention group also received 200 μg/kg of IVM as a single oral dose. Clinical improvement of baseline cough, dyspnea, and oxygen saturation was the primary outcome. The study showed faster clinical improvement of symptoms in the intervention group with a mean dyspnea duration of 2.6 days, persistent cough duration of 3.1 days, and mean hospital stay of 7.1 days, compared to 3.8, 4.8, and 8.4 days, respectively, in the control group. Additionally, the frequency of lymphopenia was significantly reduced to 3 patients in the IVM group, compared to 13 patients in the standard treatment group. Based on the results, it was concluded that the single weight-based IVM dose (200 μg/kg) could improve clinical symptoms in COVID-19 patients [53].

Another trial was conducted by Mahmud et al. between June and August 2020 in Bangladesh to investigate whether the use of IVM with doxycycline reduced the recovery duration in patients with mild-to-moderate COVID-19. The study design was a blinded, randomized, placebo-controlled, two-arm study involving 200 patients assigned to 12 mg of oral IVM with 100 mg of doxycycline combination treatment versus 200 patients assigned to placebo treatment. Both groups also received standard treatment which included acetaminophen, cough suppressants, antihistamines, vitamins, oxygen therapy if needed, low molecular weight heparin if needed, other appropriate broad-spectrum antibiotics, remdesivir, other antiviral drugs, and other drugs related to pre-existing comorbid conditions. The median time to recovery was 7 days in the group receiving IVM with doxycycline and 9 days in the placebo group. Sixty-one percent of the participants in the intervention group recovered within <7 days compared to forty-four percent in the placebo group. Additionally, a significantly lower number of participants in the intervention group remained PCR positive on day 14 and were less likely to progress to more serious disease compared to the placebo group [54].

### 7.4. Mortality Rate

Another positive role of IVM combination therapy was reported with regards to the reduction in mortality rate. A retrospective observational study was conducted by Rajter et al. on hospitalized patients with COVID-19 at four Broward Health hospitals in Florida, United States, between March and May 2020. The study included 280 patients, 173 had received IVM and 107 had not, with most of the patients receiving HCQ, azithromycin, or both. The IVM group patients received at least 200 μg/kg orally as a single dose along with the routine clinical care. Significantly lower mortality rate was observed in the IVM receiving patient (15.0%) compared to patients who did not receive IVM (25.0%), *p* = 0.03. However, no significant difference was observed in the length of stay or rate of extubating [55].

Similar results for the mortality rate were reported in Tlaxcala, Mexico, by Lima-Morales et al. who conducted a non-randomized clinical trial to evaluate the effectiveness of a multidrug-therapy (TNR4 therapy) in COVID-19-positive cases between May and September 2020. TNR4 therapy consisted of oral IVM (12 mg single dose), montelukast (60 mg on day 1 and then 10 mg from day 2 to day 21), acetylsalicylic acid (100 mg daily for one month), and azithromycin (500 mg for four consecutive days). There were 768 participants in this study divided into two groups: the TNR4 group included 481 cases who received the TNR4 therapy, and the control group included 287 cases who were self-medicated for cold and flu symptoms or had received other unspecified treatments. The investigators concluded the study by listing the major benefits of using the TNR4 therapy against COVID-19. By day 14, a significantly higher number of patients in the TNR4 group (84.4%) recovered compared to patients in the control group (58.9%). The overall probability of full patient recovery in the TNR4 group was 3.4 times greater than the control group. Moreover, the TNR4 group had a 75% lower risk of hospitalization than the control group. Mortality risk was also reduced significantly as it was 81% less in the TNR4 group. Overall, the study proved the effectiveness of the TNR4 therapy against COVID-19 [56].

Additionally, in the period between May and September 2020 in Turkey, Okumuş et al. performed a randomized, controlled, single-blinded prospective study investigating the effect of IVM addition to the treatment protocol of patients with severe COVID-19. A group of 30 patients was given oral IVM 200 μg/kg/day for 5 days alongside azithromycin, favipiravir, and HCQ. The control group consisted of 30 patients who received HCQ, azithromycin, and favipiravir, only. Following the end of the treatment period, patients were followed up for 5 days. Results showed a clinical improvement rate of 73.3% in the intervention group compared with 53.3% in the control group (*p* = 0.10). Mortality in the first group developed in six patients (20%) compared to nine patients (30%) in the control group (*p* = 0.37). The lymphocyte count in the intervention group (1698 ± 1438) was higher compared to the control group (1256 ± 710), (*p* = 0.24). The IVM-receiving group showed a more obvious reduction in serum CRP, D-dimer, and ferritin levels (*p* = 0.02, *p* = 0.03, and *p* = 0.005, respectively) compared to the control group. Results showed that clinical recovery was increased, prognostic laboratory parameters improved, and mortality rate decreased in the IVM group, even in patients with severe COVID-19. The authors also concluded that IVM should be used either as a substitute for a drug in the treatment protocol or in combination with the preexisting protocols [57]. A summary of reviewed studies on the role of IVM as part of combination therapy against COVID-19 is included in Table 3.

## 8. New Formulations of IVM Specifically Developed for COVID-19

The previously reviewed clinical studies demonstrated variable and suboptimal efficacy levels for orally administered IVM in humans against SARS-CoV-2. As a result, research scientists investigated the possibility of using other routes of administration and targeted therapy to achieve higher and optimal levels of IVM, specially through the lungs. In a study by Albariqi et al. evaluating the safety and pharmacokinetics of inhaled IVM in mice, the inhaled formulation showed promising results [58]. The improved pharmacokinetics of the inhaled formulation might be attributed to the reduction in protein binding in the systemic circulation, as well as the sustained high exposure in the respiratory tract.

Another animal study was conducted in Argentina by Errecalde et al. to assess the safety and pharmacokinetics of a novel nasal spray formulation of IVM. Since the viral entry and replication is primarily through the nasopharyngeal site followed by viral colonization at the oropharynx, the trial was conducted to assess whether a nasal formulation of IVM can attain high concentrations in the respiratory tract and consequently can be more effective, compared to oral administration. Forty healthy pigs were divided into two groups of equal size. The first group received 2 mg of oral IVM tablet. The second group received methylene blue colored nasal spray with a dose of 1 mg of IVM in 0.1 mL per puff as micro-droplets. The performance of the device during drug delivery was sufficient according to this pre-clinical study. The colored spray showed homogeneous distribution in the nasopharynx of tested pigs. Considering the high lipophilicity of IVM, the study reported high and persistent concentrations of IVM in the nasopharyngeal tissue and limited systemic absorption following nasal administration at lower doses as opposed to the oral route. According to performed safety tests, animals receiving nasal spray of IVM showed no clinical, hematological, histopathological, and serum biochemical adverse effects during the study period, compared to some minor side effects reported in the oral group [59].

Similar results were reported by Chaccour et al., utilizing nebulized ethanol-based IVM formulation in 14 Sprague-Dawley rats which received either IVM at a low dose (80–90 mg/kg), high dose (110–140 mg/kg), or ethanol only for the control group. The study found that IVM was able to achieve pharmacodynamic concentrations in the rats’ lungs without histological changes. However, the investigators highlighted safety concerns related to the ethanol vehicle and dosing regimen if a similar study was to be conducted in humans [60].

Studies investigating targeted delivery formulation were also conducted in both animals and humans. Aref et al. studied the efficacy of an IVM mucoadhesive intranasal nanosuspension spray in reducing the upper respiratory symptoms in mild COVID-19 cases. The clinical trial took place in Qena University Hospital, Egypt. It included 114 patients diagnosed with mild COVID-19 that were divided into two groups. Group A consisted of 57 patients and received the Egyptian protocol for mild COVID-19 with IVM nanosuspension twice daily, while Group B consisted of 57 patients who received the Egyptian protocol for mild COVID-19 alone. Clinical manifestations, hematological, biochemical parameters as well as two consecutive negative nasopharyngeal swabs for COVID-19 were used to evaluate the patients. The results showed that 54 patients of group A (94.7%) achieved 2 consecutive negative PCR nasopharyngeal swabs compared to only 43 patients (75.4%) from Group B (*p* = 0.004). The negative PCR results were obtained significantly faster in group A (8.3 ± 2.8 days) compared to group B (12.9 ± 4.3 days) (*p* = 0.0001). The duration of clinical manifestations including fever, anosmia, cough, and dyspnea were also significantly shorter in group A compared to group B, excluding gastrointestinal symptoms. In conclusion, the study supports the local use of mucoadhesive IVM nanosuspension in mild COVID-19 cases [61].

In another observational study conducted in Osaka, Japan, IVM was administered as a nasal formulation to COVID-19 patients under mechanical ventilation. Eighty-eight ICU COVID-19 patients, who were on mechanical ventilation, were divided into two groups: the IVM group and the control group. The IVM group consisted of 39 patients who received nasal IVM 200 µg/kg within 3 days of ICU admission. The investigated primary outcome was the ventilation free days (VFD) measured 4 weeks post admission. VFD are days in which the patients are alive and free from mechanical ventilation. The secondary outcomes included gastrointestinal complications, diarrhea, and regurgitation that were measured 4 weeks post admission. The study results showed a significantly higher VFD and a significantly lower recurring frequency of gastrointestinal complications, diarrhea, and regurgitation in the IVM group compared to the control group. Additionally, the mortality rate during the ICU stay was significantly lower in the IVM group compared to the control group, amounting to 0% and 16.03%, respectively. The authors concluded that nasal IVM had a beneficial influence on VFD and gastrointestinal complications, and there is room for more investigation for IVM use in COVID-19 patients [62].

## 9. IVM Safety Concerns

With the greater attention and subsequently increased use of IVM since its association with COVID-19, it is important to consider what implications this might carry in the future. Although the safety of IVM was one of the highest amongst the newly repurposed drugs [63], it is still vital to understand how this translates in practice with its current growing and often misinformed use.

In a study conducted by Dicks et al., the long-term effects of IVM on the gut microbiota and dysbiosis were investigated. It was reported that prolonged oral use of IVM could lead to an imbalance in the oral microbiome. The oral microbiome is an essential constituent in developing the gut microbiota. Gut dysbiosis is linked with irritable bowel syndrome (IBS), enterocolitis, and diarrhea. Furthermore, an abnormal gut microbiome is associated with neurological and psychiatric diseases due to the affected gut-brain access [64]. IVM bacteriostatic activity evidenced by time-kill kinetics was also demonstrated by Ashraf et al. [65]. Thus, the repurposing of IVM in COVID-19 and its prolonged use could potentially result in gut dysbiosis.

Gut dysbiosis was not the only concern of IVM toxicity. The Oregon Poison Center reported patients suffering from episodes of ataxia, confusion, hypotension, and seizures as well as gastrointestinal tract distress. The resultant toxicity was observed in patients receiving high frequent doses of IVM up to once every other day [66]. Therefore, the clinical use of IVM in COVID-19 patients must be investigated further with more focus on its safety profile at the proposed dosing regimen to fully understand what the future of IVM use in COVID-19 patients might look like.

## 10. Discussion and Conclusions

The recent global pandemic of COVID-19 has elicited a wave of studies and research investigations with the goal of identifying an effective and safe treatment. IVM was one of the explored drugs, although its exact mechanism of action against SARS-CoV-2 was not fully understood. The scope of conducted research studies/clinical trials included understanding its mechanism of action, determining its effectiveness in the treatment/prophylaxis against COVID-19, as well as exploring the advantage of targeted delivery and novel IVM formulations.

Studies that aimed to explore the mechanism of action of IVM against COVID-19 reported several potential mechanisms, including the inhibitory effect on RNA replication, obstruction of binding to the receptor sites, and the immunomodulatory effect. However, all these studies were conducted in either cell lines or animals, but not in humans.

Although reports from several clinical trials do not show that IVM could be the wonder drug, it was deemed that for the treatment/prophylaxis of COVID-19, there is still room for its repurposed use. As observed globally and across many studies, IVM showed moderate benefit in mild COVID-19 cases when utilized alone or as a part of combination treatment. These benefits include a shorter duration of treatment, faster negative PCR results, reduction of viral load and earlier viral clearance. On the other hand, it did not show considerable impact regarding symptom resolution when used alone as opposed to significant improvements when used as a combination therapy. Additionally, decrease in mortality rate was reported for combination therapies that included IVM. Further, it was evident that IVM played an effective role when utilized for prophylaxis. It was noticed that studies investigating the prophylactic potential of IVM included a higher number of subjects and were mostly conducted retrospectively.

Researchers have developed and studied targeted delivery formulations of IVM through the nasal route for COVID-19 in both animals and humans and reported improved pharmacokinetics, higher concentrations in the nasopharynx/lungs, and better resolution of clinical manifestations. With these promising outcomes, it would be reasonable to assume that another door could be opened for the role of IVM in COVID-19. Some safety concerns regarding prolonged IVM use revolved around its impact on the gut microbiome and the toxicity resulting from high and frequent dosing. Therefore, IVM use for COVID-19 has the merit to be explored further on a larger scale with more emphasis on its safety.

## Figures and Tables

**Table 1:** Summary of the reviewed studies on the potential of IVM in the treatment of COVID-19.

Date	Country	Study Design	IVM Dose	# of Patients	Main Outcomes	References
May–November 2020	Nigeria	Randomized, controlled, double-blinded two-groups trial	6 mg IV twice a week or 12 mg IV twice a week	62	Significant shortened duration of treatment	[35]
July–September 2020	India	Randomized, controlled, double-blinded trial	Single oral dose, 24 mg, or 12 mg	125	Insignificant effect on time to PCR negativity	[36]
May–September 2020	Argentina	Randomized, controlled, outcome-assessor blinded trial	600 μg/kg/day as oral tablet for 5 days	30	Significant viral load reduction	[37]
September–November 2020	Lebanon	Randomized, controlled, parallel groups trial	Single oral weight-based dose (45–64 kg, 65–84 kg, ≥85 kg received 9 mg, 12 mg or 150 μg/kg respectively)	100	Significant viral load reduction	[38]
July–September 2020	Spain	Randomized, controlled, double-blinded trial	Single oral dose (400 μg/kg)	24	Significant faster recovery from hyposmia/anosmia, insignificant viral load reduction	[39]
	Bangladesh	Randomized, double-blinded, placebo-controlled trial	Oral 12 mg/day for 5 days, or oral 12 mg as a single dose +200 mg doxycycline on day 1, then doxycycline 100 mg every 12 h for 4 days.	72	Significant earlier viral clearance, insignificant clinical symptoms recession	[40]
April–August 2020	Mexico	Randomized, controlled, double-blinded trial	Single oral weight-based dose (12 mg for <80 kg, 18 mg for >80 kg)	106	Insignificant effect on hospitalization duration, respiratory deterioration, or death	[41]
March–October 2020	Egypt	Randomized, open-label parallel-groups trial	Single oral dose (12 mg/day) for 3 days.	164	Insignificant reduction of hospital stays and mortality rates	[42]
July–December 2020	Colombia	Randomized, double-blinded trial	300 μg/kg/day orally (as a solution) for 5 days	400	Insignificant reduction of time to symptom resolution	[43]
August 2020–February 2021	Argentina	Randomized, double-blinded, placebo-controlled trial	Weight-based (≤80 kg, >80 to ≤110 kg, and >110 kg received 12 mg, 18 mg or 24 mg respectively) for 2 consecutive days	501	Insignificant effect on hospitalization prevention	[44]

**Table 2:** Summary of the reviewed studies on the potential of IVM in the prophylaxis against COVID-19.

Date	Country	Study Design	IVM Dose	# of Subjects	Main Outcomes	References
June–July 2020	Egypt	Randomized, open label-controlled study	Oral dose of 200–300 μg/kg, on the 1st and 3rd days.	304	2-days delay in symptoms development	[45]
January 2022	Brazil	Observational, prospective study	200 μg/kg for 2 consecutive days	159,561	Significant reduction in the infection rate (44%), mortality rate (68%), and hospitalization rate (67%)	[46]
October 2020	54 African countries	Retrospective study	Not specified	1,336,943,343	Significantly lower COVID-19 infection and mortality rates	[47]
June–July 2020	Dominican Republic	Observational retrospective cohort study	Weekly oral dose of 200 μg/kg for 4 weeks	542	Significant prophylaxis against the infection	[48]
September–October 2020	India	Hospital-based matched case-control study	2 oral doses of 300 μg/kg, 72 h apart	372	Reduced COVID-19 infection (73%)	[49]
October 2020	Worldwide	Retrospective study	Not specified	Not specified (all COVID-19 incidences as of 5 June 2020)	Significantly lower incidence of COVID-19	[50]

**Table 3:** Summary of the reviewed studies on the potential of IVM as part of combination therapies in COVID-19.

Date	Country	Study Design	IVM Dose/Other Therapeutics	# of Patients	Main Outcomes	References
May–June 2020	Bangladesh	Randomized, controlled study	200 μg/kg as a single oral dose on day 1 + doxycycline 100 mg twice daily for 10 days. or HCQ 400 mg on day 1 then 200 mg twice daily + azithromycin 500 mg/day for 5 days	116	IVM + Doxycycline was superior to HCQ + Azithromycin	[51]
May–October 2020	Egypt	Non-randomized study	Weight-based oral dose every 72 h (60–90 kg, 90–120 kg, >120 kg received 200–400 μg/kg, 300–400 μg/kg, and 30 mg respectively), 500 mg nitazoxanide every 6 h, ribavirin 400 mg every 6 h	113	The use of the triple therapy cleared COVID-19 virus within a shorter period	[52]
May–July 2020	Iran	Randomized, double-blinded study	200 μg/kg as a single oral dose/HCQ and/or lopinavir/ritonavir	69	Faster clinical improvement of symptoms	[53]
June–August 2020	Bangladesh	Randomized, blinded, placebo-controlled study	12 mg oral dose/doxycycline (100 mg)	400	Significantly lower number of participants remained PCR positive on day 14	[54]
March–May 2020	United States	Retrospective, observational study	200 μg/kg orally as a single dose/HCQ, azithromycin	280	Significantly lower mortality rate, insignificant reduction in the length of stay or rate of extubating	[55]
May–September 2020	Mexico	Non-randomized study	12 mg single oral dose/montelukast (60 mg on day 1 and then 10 mg from day 2 to day 21), acetylsalicylic acid (100 mg daily for one month), and azithromycin (500 mg for four consecutive days)	768	Higher full recovery in the TNR4, 75% lower risk of hospitalization, reduced mortality risk	[56]
May–September 2020	Turkey	Randomized, controlled, single-blinded study	Oral 200 μg/kg/day for 5 days/azithromycin, favipiravir, and HCQ	60	Clinical recovery is increased, prognostic laboratory parameters improved, mortality rate decreased	[57]

## Data Availability

Data sharing not applicable.
