# Correction: Awad et al. Repurposing Potential of the Antiparasitic Agent Ivermectin for the Treatment and/or Prophylaxis of COVID-19. *Pharmaceuticals* 2022, *15*, 1068

**DOI:** 10.3390/ph17081100

**Published:** 2024-08-22

**Authors:** Hoda Awad, Basmala Hassan, Sara Dweek, Yasmeen Aboelata, Mutasem Rawas-Qalaji, Iman Saad Ahmed

**Affiliations:** 1 Department of Pharmaceutics & Pharmaceutical Technology, College of Pharmacy, University of Sharjah, Sharjah 27272, United Arab Emirates; u19104592@sharjah.ac.ae (H.A.); u17100019@sharjah.ac.ae (B.H.); u17103078@sharjah.ac.ae (S.D.); u17102409@sharjah.ac.ae (Y.A.); mqalaji@sharjah.ac.ae (M.R.-Q.); 2 Research Institute of Medical and Health Sciences, University of Sharjah, Sharjah 27272, United Arab Emirates

## Text Correction

The following paragraph, “Coinciding but insignificant findings with regards to the time to negative PCR were established by Pott-Junior et al. in a randomized open-label study conducted at Federal University of São Carlos, Brazil, in a group of 32 mild COVID-19 patients who received standard of care (SOC) alone, or SOC and oral IVM (3 different groups received the following doses: 100 μg/kg, 200 μg/kg and 400 μg/kg). The study did not report any serious adverse events in the SOC with IVM group. Patients receiving IVM required a shorter time to obtain two consecutive negative PCR result in a dose-dependent manner [52]”, should be ignored since the manuscript was retracted and is no longer part of the scientific record due to a lack of sufficient detail for some of the methods and approaches in the experimental design [1].

A correction has been made to delete this paragraph and the reference included in it. Additionally, the related content in Table 3 has been deleted, and all the other reference numbers have been reordered accordingly.

The authors state that the scientific conclusions are unaffected. This correction was approved by the Academic Editor. The original publication [2] has also been updated.
